# Group treatment experience in a brief psychiatry hospitlization unit

**DOI:** 10.1192/j.eurpsy.2021.1326

**Published:** 2021-08-13

**Authors:** R. Pinilla, U. Aragones, B. Ordoñez, A. Sotillos, A. Hernández Mata

**Affiliations:** 1 Psiquiatria, Hospital de Getafe, Madrid, Spain; 2 Psiquiatría, Hospital Universitario de Getafe, Getafe, Spain

## Abstract

**Introduction:**

Joseph Pratt, a sanatorium doctor, at the beginning of the 20th century began to organize groups of patients in order to transmit information about their illness, observing that those who came had a better evolution. In the twenties, Jacob L. Moreno, would make the leap towards mental health, transferring the group format to the treatment of mental disorders. At the same time, Lazell and Marsh began to carry out psychoeducational groups with admitted schizophrenic patients.

**Objectives:**

Present experience of a psychotherapeutic group in a brief psychiatry hospitalization unit.

**Methods:**

Non-directional, voluntary group, with daily frequency and 30 minutes duration. Between 8-15 patients participated. Participation in the group required compliance with 2 rules: respecting word turns and speaking from one’s own experience. The sessions were organized in three parts, 1. Opening of the group: the rules are remembered and we welcome new patients. 2. Group: dialogue between patients 3. Group closure: summary of the session and dismissal of discharge patients.

**Results:**

The following topics were addressed: - The experience of admission; traumatic vs restorative. - The difficulties they expected to encounter after discharge. - Aspects related to family bonding, between equals and couples. As difficulties we find: - The heterogeneity in the symptoms of the patients. - Voluntary participation in the group. - Conflicts reactive to non-compliance with the rules.

**Conclusions:**

Group therapies in brief hospitalization units have great therapeutic potential.

**Conflict of interest:**

No significant relationships.

